# Scar Pregnancy: A Rare, but Challenging, Obstetric Condition

**DOI:** 10.3390/jcm12123975

**Published:** 2023-06-11

**Authors:** Roberta Granese, Ferdinando Antonio Gulino, Giosuè Giordano Incognito, Canio Martinelli, Stefano Cianci, Alfredo Ercoli

**Affiliations:** 1 Unit of Gynecology and Obstetric, Department of Biomedical and Dental Sciences and Morpho Functional Imaging, University Hospital “G. Martino”, 98100 Messina, Italy; 2 Unit of Gynecology and Obstetric, Department of Human Pathology of Adults and Developmental Age, University Hospital “G. Martino”, 98100 Messina, Italy; docferdi@hotmail.it (F.A.G.); caniomartinelli.md@gmail.com (C.M.); stefanoc85@hotmail.it (S.C.); alfercoli@yahoo.it (A.E.); 3 Department of Gynecology and Obstetric, University Hospital “San Marco”, 95100 Catania, Italy; giordanoincognito@gmail.com

Scar pregnancy is a rare and potentially life-threatening condition that occurs when an embryo implants and grows within a previous cesarean section scar or other uterine scars. This unique form of ectopic pregnancy poses significant diagnostic and management challenges for healthcare providers. Scar pregnancies are associated with an increased risk of uterine rupture, massive hemorrhage, and other complications, underscoring the importance of early detection and appropriate management strategies [1]. Scar pregnancies are rare, accounting for less than 1% of all ectopic pregnancies. They typically arise in women with a history of prior uterine surgery, including cesarean sections, myomectomies, or other procedures that create a uterine scar [2]. The embryo implants within the scar tissue, instead of the normal uterine cavity, leading to an abnormal pregnancy with potential risks to maternal health. Diagnosing scar pregnancy can be challenging due to its rarity and varied clinical presentation. Early ultrasound evaluation is crucial for detecting this condition. Transvaginal ultrasound plays a vital role in visualizing the gestational sac within the cesarean scar and differentiating it from other types of ectopic pregnancies [3]. Serial monitoring of serum beta-human chorionic gonadotropin (β-hCG) levels and close observation of clinical symptoms are also essential for accurate diagnosis. Scar pregnancy poses unique management considerations due to the increased risk of complications associated with the previous uterine scar. Several treatment options exist, including medical management with methotrexate [4], surgical interventions [5], such as dilation and curettage, or a combination of both. The choice of management depends on factors, such as the patient’s hemodynamic stability, gestational age, and desire for future fertility [6,7,8]. Individualized care and a multidisciplinary approach, involving obstetricians, radiologists, and reproductive specialists, are crucial for optimizing outcomes. Scar pregnancy carries a higher risk of uterine rupture than other ectopic pregnancies. The thin and weak scar tissue may be unable to withstand the growing pregnancy, resulting in uterine rupture and life-threatening hemorrhage [9]. Prompt intervention and careful monitoring are essential to prevent these complications. In cases where scar rupture occurs, emergency surgical intervention, including hysterectomy, may be necessary to ensure the mother’s wellbeing. Apart from the physical risks, scar pregnancy can also have a significant psychological impact on the affected women and their families. The emotional stress associated with the potential loss of fertility, the fear of uterine rupture, and the anxiety surrounding subsequent pregnancies can be overwhelming. Appropriate counseling, emotional support, and access to mental health services are crucial components of comprehensive care for women facing scar pregnancies. In conclusion, scar pregnancy is a rare obstetric condition that requires early diagnosis and individualized management to minimize potential risks to maternal health. With advancements in ultrasound technology and increased awareness among healthcare providers, timely detection of scar pregnancies is becoming more achievable. However, further research is needed to refine diagnostic and management approaches and to optimize outcomes for affected women. By combining medical expertise, multidisciplinary collaboration, and comprehensive support, we can navigate the challenges of scar pregnancies and provide the best possible care for women facing this complex obstetric condition.
